# Development of a Nafion/MWCNT-SPCE-Based Portable Sensor for the Voltammetric Analysis of the Anti-Tuberculosis Drug Ethambutol

**DOI:** 10.3390/s16071015

**Published:** 2016-06-30

**Authors:** Rosa A. S. Couto, Maria Beatriz Quinaz

**Affiliations:** LAQV/REQUIMTE, Department of Chemical Sciences, Faculty of Pharmacy, University of Porto, 4050-313 Porto, Portugal; rcouto@ff.up.pt

**Keywords:** carbon nanotube, electrochemistry, ethambutol, pharmaceutical analysis, screen-printed electrode, sensor, tuberculosis

## Abstract

Herein we describe the development, characterization and application of an electrochemical sensor based on the use of Nafion/MWCNT-modified screen-printed carbon electrodes (SPCEs) for the voltammetric detection of the anti-tuberculosis (anti-TB) drug ethambutol (ETB). The electrochemical behaviour of the drug at the surface of the developed Nafion/MWCNT-SPCEs was studied through cyclic voltammetry (CV) and square wave voltammetry (SWV) techniques. Electrochemical impedance spectroscopy (EIS) and scanning electron microscopy (SEM) were employed to characterize the modified surface of the electrodes. Results showed that, compared to both unmodified and MWCNTs-modified SPCEs, negatively charged Nafion/MWCNT-SPCEs remarkably enhanced the electrochemical sensitivity and selectivity for ETB due to the synergistic effect of the electrostatic interaction between cationic ETB molecules and negatively charged Nafion polymer and the inherent electrocatalytic properties of both MWCNTs and Nafion. Nafion/MWCNT-SPCEs provided excellent biocompatibility, good electrical conductivity, low electrochemical interferences and a high signal-to-noise ratio, providing excellent performance towards ETB quantification in microvolumes of human urine and human blood serum samples. The outcomes of this paper confirm that the Nafion/MWCNT-SPCE-based device could be a potential candidate for the development of a low-cost, yet reliable and efficient electrochemical portable sensor for the low-level detection of this antimycobacterial drug in biological samples.

## 1. Introduction

Tuberculosis (TB), a contagious multisystemic disease caused by *Mycobacterium tuberculosis* (Mtb), is one of the oldest infectious diseases affecting humankind [1]. Nevertheless, TB is still a major global public health problem, currently considered the leading cause of death from infectious disease in the world [2]. According to the World Health Organisation’s global TB report of 2015, in 2014 TB caused 1.5 million of deaths and infected 9.6 million people, while 480 million infected individuals developed multidrug-resistant TB (MDR-TB) [2]. Indeed, the widespread existence of MDR strains of Mtb, combined with the significant increasing of HIV incidence in the last years, makes TB an emergent worldwide issue [3]. 

Currently, there are more than twenty drugs being used to treat TB, differing in their efficiency, drug class, potency and usability [4]. First-line anti-TB drugs include isoniazid, rifampicin, pryrazinamide and ethambutol (ETB), which are administrated in a multi-drug combination strategy [5]. Such complex pharmacological procedures are frequently associated with numerous side effects and poisoning incident reports and to inappropriate or incorrect use of anti-TB drugs, contributing to the growing MDR-TB incidence [3]. In this context, there is a strong need for extensive analysis of anti-TB drugs, being essential to design and develop a portable, sensitive and reliable sensor for antimycobacterium drugs control [6]. Notwithstanding the existence of several analytical methods for the detection of anti-TB drugs, such as high performance liquid chromatography (HPLC) [7], liquid chromatography/tandem mass spectrometry (LC/MS) [8,9,10], micellar electrokinetic capillary chromatography (MEKC) [11], chemiluminescence [12,13,14], fluorimetry [15,16], colorimetry [17], titrimetry [18] and voltammetry [19,20], there is still a strong need for the development of portable devices for effective and rapid analysis of these compounds. Among the reported methods, electrochemical detection is recognized as one of the most promising analytical techniques due to its simplicity, cost-effectiveness and potential for in-situ drug monitoring [6,21]. Indeed, there has been an increasing trend towards miniaturization of electrochemical sensors and biosensors over the past decades [22,23,24]. Particularly, the development of electrochemical sensors based on the use of screen-printed electrodes (SPEs) is currently undergoing a widespread growth [25,26,27,28]. Among the several advantages of SPEs-based devices, their great versatility lies in the variety of materials that can be employed in the production of the working electrodes (WEs) [29] and in the wide range of ways in which the WEs may be modified, resulting in an enhancement of their functions and applications [25,30]. Carbon is the material most often applied in the construction of SPEs due to its low background current, readily renewable surface, wide potential window, inexpensiveness, versatility and chemical inertia [31]. Particularly, carbon nanotubes (CNTs) are especially attractive electrode nanomaterials with high electrical conductivity, significant mechanical strength and unique electrocatalytic properties [32] that can be used to modify the carbon WEs surface and enhance the sensitivity and selectivity to the electrochemical system [33,34]. Furthermore, the SPEs surface modification with polymers, such as Nafion, can provide additional advantages [35,36]. Nafion is a sulfonated tetrafluoroethylene based anionic copolymer with cationic exchange properties that allows the easy production of modified electrodes [37,38]. Its role is frequently associated to the enhancement of the analytical signal intensity and minimization of the interference of electroactive species. In fact, its physico-chemical properties, such as good electrical conductivity, high partition coefficients of many redox compounds and biocompatibility, play an important role on the protection of the WEs surface, minimizing the interferences influence [35,37].

**Scheme 1:**
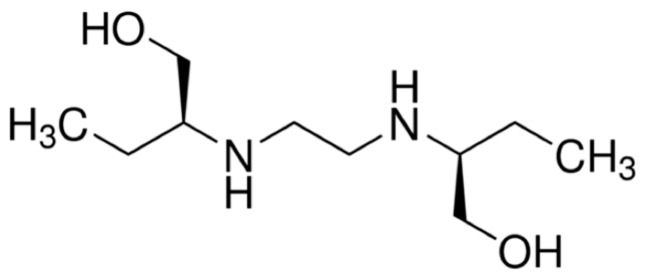
Chemical structure of the antimycobacterial drug ETB.

In this paper, SPEs with carbon WEs (SPCEs) were modified with multi-walled CNTs (MWCNTs) and Nafion and applied to the study of the voltammetric behaviour of the first-line anti-TB drug ETB (Scheme 1). ETB is an organic cation at physiological pH [39] particularly helpful in the treatment of MDR-TB that has been slightly electrochemically studied [6,40,41]. Here, the synergistic effect of the Nafion coating of MWCNT-SPCEs, resulted from the electrocatalytic properties of both MWCNTs and Nafion and the predicted selectivity of the anionic Nafion polymer to the positive charged ETB molecule is explored. 

## 2. Materials and Methods

### 2.1. Chemicals

All solutions were prepared with water from a Milli-Q system (conductivity ≤ 0.1 μS·cm^−1^) and chemicals of analytical reagent grade quality that were not exposed to any further purification. 

Nafion perfluorinated resin solution 5 wt % in mixture of lower aliphatic alcohols and water, with 45% water was purchased from Sigma-Aldrich (St. Louis, MO, USA). Working solutions of Nafion were prepared by suitable dilutions with deionised water.

Supporting electrolytes of citrate-phosphate buffer solutions (CPBS) with pH values of 2.0, 4.0 and 7.4 and of borax-NaOH buffer solutions pH values of 9.0 and 11.0 were used.

ETB dihydrochloride (C_10_H_26_Cl_2_N_2_O_2_) was purchased from Sigma-Aldrich. Stock solutions of ETB were daily prepared by dissolving of suitable quantities of the drug in deionised water. Working standard solutions were prepared by suitable dilutions of the stock solutions with CPBS pH 7.4. All experiments were performed at 25.0 ± 0.5 °C. 

### 2.2. Apparatus

Electrochemical measurements, namely cyclic voltammetry (CV) and square wave voltammetry (SWV), were performed with a PC-controlled 910 PSTAT mini potentiostat (Metrohm, Herisau, Switzerland) controlled by a PSTAT software for data acquisition and experimental control. Both unmodified- and MWCNT-SPCEs were purchased from DropSens (Llanera, Asturias, Spain), comprising a 3 mm diameter WE, a carbon counter electrode and a silver pseudoreference electrode (ref. DRP-110 and DRP-110CNT, respectively). The ceramic substract of the SPEs had the general dimensions of 33 mm length, 10 mm width and 0.5 mm height and the electric contacts were made of silver. The electrochemical measurements were performed in five different WEs: unmodified-SPCEs, Nafion-SPCE, MWCNT-SPCEs, Nafion/MWCNT-SPCEs and Nafion/graphene-SPCE. The electrode cable and the connector for PSTAT were supplied by Metrohm (Herisau, Switzerland). All electrochemical measurements were performed at 25 ± 0.5 °C.

The electrodes surfaces were examined using a high resolution scanning electron microscope with X-ray microanalysis: QUANTA 400 FEG ESEM/EDAX PEGASUS X4M (FEI, NE Dawson Creek Drive, Hillsboro, OR, USA) at the CEMUP Laboratory (University of Porto, Porto, Portugal).

Electrochemical impedance spectroscopy (EIS) studies were performed using an Autolab PGSTAT204 potentiostat/galvanostat expanded with a FRA32M EIS module (Metrohm) and NOVA 1.10.1.9 software (Metrohm Autolab, KM Utrecht, The Netherlands) for data acquisition.

### 2.3. SPCEs Modification

The disposable SPEs purchased from DropSens were prepared through screen-printing of the three electrodes inks on an inert ceramic substrate and exposed to a high-temperature curing step and a protective ink coating in order to insulate the conductive tracks from the electrodes. For the surface modification of the WEs with Nafion, Nafion solutions of different concentrations (0.0125%, 0.0250% and 0.050%) were prepared by dilution of the Nafion perfluorinated resin solution 5 wt % in deionised water; 10 μL of Nafion solution were dropped over the WEs surface of unmodified-, MWCNT- and graphene-SPCEs, followed by a dry step at 75 °C for 2 h for complete solvent evaporation, ensuring the batch-to-batch repeatability of SPCE modification. The prepared electrodes were stored at room temperature and washed with deionised water before the first measurement and whenever it was necessary. Each measurement was performed in triplicate with the same electrode and a new electrode was used for each experiment, unless specified otherwise. No further treatment steps were used.

### 2.4. Analytical Procedure

The electrochemical behaviour of ETB was investigated at the surface of five different SPCEs: unmodified-, Nafion-, MWCNT-, Nafion/MWCNT- and Nafion/graphene-SPCEs by CV and SWV. Measurements were performed with a 100 μL drop of ETB solutions in CPBS pH 7.4, covering the surface of the screen-printed three-electrode system. In the case of biological samples, dilutions of 1:10 (urine) and 1:2 (serum) were performed with CPBS pH 7.4. No additional pre-treatment steps were required.

## 3. Results and Discussion

### 3.1. Optimization of the Modifier

**Figure 1:**
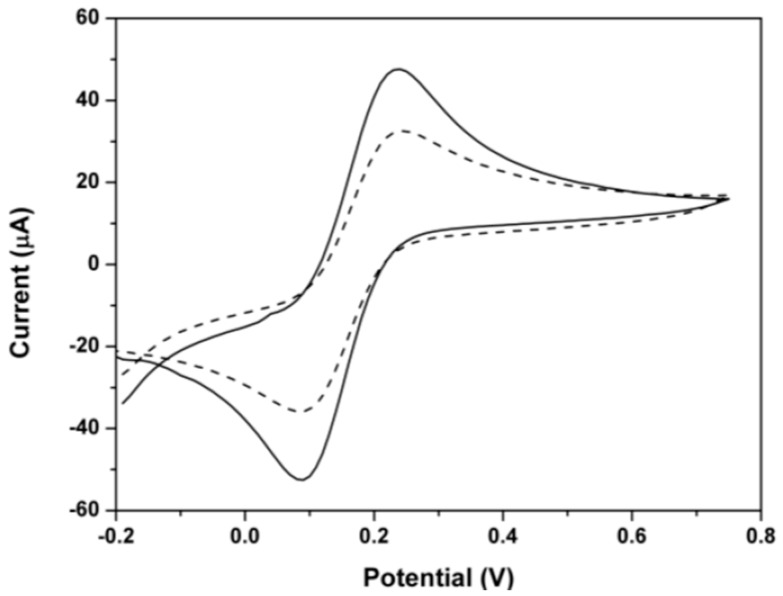
Cyclic voltammograms of a 2 mM [Fe(CN)_6_]^3−/4−^ at the surface of a MWCNT-SPCE (solid line) and a Nafion/MWCNT-SPCE (dash line). Scan rate: 100 mV·s^−1^.

CV preliminary studies were developed in the presence of a 2.0 mM [Fe(CN)_6_]^3−/4−^ solution in PBS pH 7.4 to evaluate the electrochemical response of the Nafion-modified electrodes. Figure 1 depicts the comparison of the cyclic voltammograms of the redox probe [Fe(CN)_6_]^3−/4−^ obtained at the surface of a MWCNT-SPCE and a Nafion/MWCNT-SPCE, between −200 and 750 mV. The sigmoidal curves obtained at both surfaces indicates the hemispherical diffusion process of [Fe(CN)_6_]^3−/4−^ towards the WE surface, which is in agreement with the behaviour reported in literature [42].

CV results point to a decreasing in both [Fe(CN)_6_]^3−/4−^ anodic and cathodic peak currents (*I*_pa_ and *I*_pc_, respectively) at the surface of Nafion/MWCNT-SPCE, compared to MWCNT-SPCE, which is attributed to an increased electron transfer resistance as a consequence of the addition of the Nafion polymer layer [25]. In addition, giving the anionic characteristics of the redox system used, the current reduction observed can be explained by the repulsion between such species and the anionic polymer, corroborating its presence at the surface of the MWCNT-SPCEs. 

**Figure 2:**
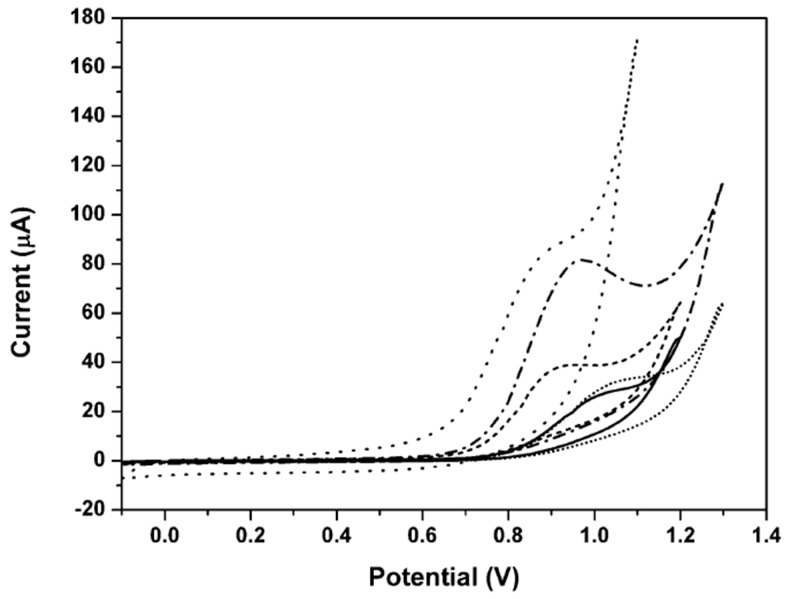
Cyclic voltammograms of a 0.14 mg/μL ETB solution in CPBS pH 7.4 at the surface of unmodified-SPCE (solid line), Nafion-SPCE (short dot line), MWCNT-SPCE (short dash line), Nafion/MWCNT-SPCE (dash dot line) and Nafion/graphene-SPCE (dot line). Scan rate: 100 mV·s^−1^.

In order to evaluate the electrochemical effect of the modifier materials and the potential application of the Nafion modified-electrodes to the sensing of ETB, the voltammetric behaviour of the drug was studied at the surface of five different SPCEs: (i) unmodified-SPCE; (ii) Nafion-SPCE; (iii) MWCNT-SPCE; (iv) Nafion/MWCNT-SPCE; and (v) Nafion/graphene-SPCE by CV in CPBS pH 7.4. Figure 2 depicts the cyclic voltammograms of a 0.14 mg/μL ETB solution obtained at the surface of the five different WEs. ETB cyclic voltammograms were compared with the one obtained in CPBS pH 7.4 (experimental blank) that showed a current close to zero, ranging from −100 to 1300 mV.

The CV results point to different interactions of the electroactive drug with the electrode surface according to the composition of the modifier applied. As can be observed in the clyclic voltammograms, the analytical signal of ETB at the surface of the unmodified-SPCE is poorly resolved, being its intensity drastically low compared with the anodic peak currents obtained to the ETB oxidation at the modified surfaces with carbon nanomaterials, such as graphene- and MWCNT-SPCEs. It can be also observed that the MWCNTs coating, either in the presence or absence of Nafion, significantly increases the anodic peak current and the definition of the oxidation wave of the analyte. In addition, the CV results show that the modification of the SPCE just with Nafion slightly increases the electrochemical signal, being the influence of the polymer remarkably higher in the presence of the carbon nanomaterials. This observation is in agreement with the expected synergy between the catalytic properties of carbon nanomaterials and Nafion, which is related to the great interaction of the protonated drug and the negatively charged copolymer applied. Furthermore, notwithstanding the intensity steps obtained to Nafion/graphene- and Nafion/MWCNT-SPCEs are very similar, it can be seen that there is a much better definition in the case of the ETB anodic wave obtained with the SPCEs modified with Nafion and MWCNTs, which were selected for the following studies. These features undoubtedly validate the advantages resulted from the simultaneous use of MWCNTs, which are high conductive nanomaterials, and the anionic polymer Nafion that has also a great affinity for ETB at pH 7.4, indicating that the Nafion/MWCNT-SPCEs can be exploited as electrochemical sensors for ETB detection. Moreover, in all instances, the cyclic voltammograms of ETB show a single anodic signal, suggesting that the drug undergoes an irreversible oxidation process at the surface of the different electrodes.

**Figure 3:**
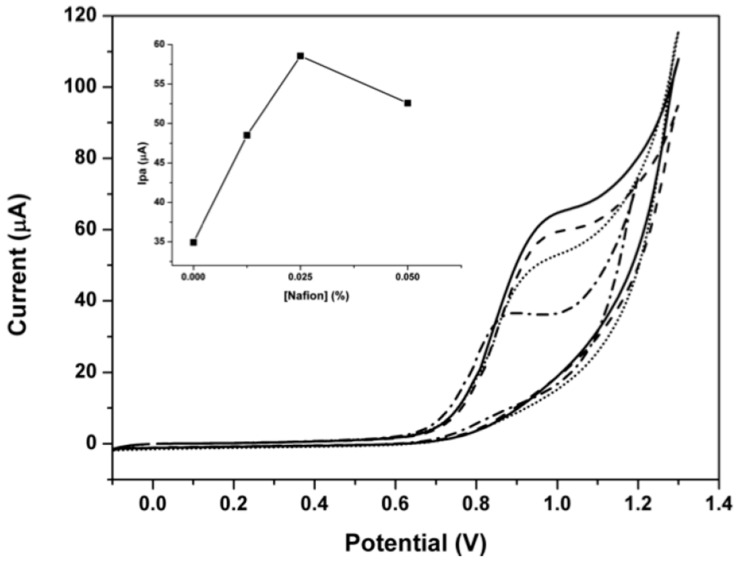
Cyclic voltammograms of a 0.08 mg/μL ETB solution in CPBS pH 7.4 at the surface of a MWCNT-SPCE (dash dot line), 0.0125% Nafion/MWCNT-SPCE (short dot line), 0.0250% Nafion/MWCNT-SPCE (solid line) and 0.0500% Nafion/MWCNT-SPCE (dash line).

In order to optimimize the electrodes coating procedure, several concentrations of Nafion solutions were used. The performance of the obtained modified electrodes was evaluated through CV by means of *I*_pa_ for a 0.08 mg/μL ETB solution in CPBS pH 7.4. Figure 3 shows ETB cyclic voltammograms obtained at the surface of MWCNT-SPCEs modified with 0.0125%, 0.0250% and 0.0500% Nafion solutions. It was found that regardless the concentration of the modifier solution, the charge transfer is facilitated in the presence of the polymer compared to the MWCNT-SPCEs without Nafion. The changes in *I*_pa_ and anodic peak potential (*E*_pa_) values observed for ETB oxidation on MWCNT-SPCEs modified with different concentrations of Nafion are attributed to the different accessibility of the drug within the film to the electrode surface [16].

In fact, the concentration of the Nafion solution determines the polymer film thickness, which influences the amount of drug that is transferred to the electrode surface during the oxidation process, consequently defining the sensitivity and repeatability of the developed electrodes [36]. Based on this, the lower *I*_pa_ obtained for 0.0500% Nafion/MWCNT-SPCEs is attributed to the formation of a thicker Nafion film that probably hampers the diffusion of ETB to the electrode surface.

As it can be confirmed in the inset of Figure 3, the highest *I*_pa_ value was obtained for the 0.0250% Nafion/MWCNT-SPCE. A well-defined oxidation signal, characteristic of a diffusion-controlled electron transfer process, was observed at the surface of 0.025% Nafion/MWCNT-SPCEs, demonstrating that Nafion can effectively increase the electroanalytical signal of ETB due to its electric properties and its affinity to this antimycobacterial drug. This optimized device was chosen and applied in the following experiments.

### 3.2. Characterization of the Nafion/MWCNT-SPCE Surface

**Figure 4:**
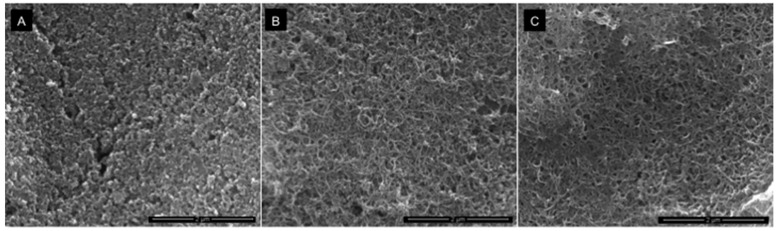
SEM images of unmodified-SPCE (**A**), MWCNT-SPCE (**B**) and Nafion/MWCNT-SPCE (**C**) surfaces with a gas secondary electron detector; 50,000 magnification.

The surface morphology of the SPCEs’ surface was studied by scanning electron microscopy (SEM). Unmodified-, MWCNT- and Nafion/MWCNT-SPCEs’ surfaces can be seen in the scanning electron micrographs in Figure 4A–C, respectively. The differences between the unmodified- and the MWCNT-SPCEs’ surfaces are evident, with highly ordered and well-defined MWCNTs, indicating that the carbon electrode was fully covered by CNTs, which are homogeneously distributed throughout the carbon WE surface. Figure 4C shows the surface coverage of a 0.025% Nafion/MWCNT-SPCE with a typical spaghetti-like reticular film, being the uniform distribution of Nafion/MWCNTs through the electrode even more pronounced, confirming the efficacy of the proposed coating procedure.

**Figure 5:**
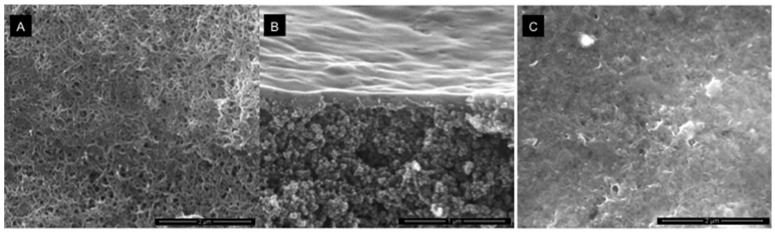
SEM images of a Nafion/MWCNT-SPCE after 12 (**A**, surface), 20 (**B**, cross section) and 50 (**C**, surface) measurements with a gas secondary electron detector; (**A**,**C**) 50,000 magnification, (**B**) 100,000 magnification.

Furthermore, SEM images of Nafion/MWCNT-SPCEs’ surface after 12, 20 and 50 measurements are shown in Figure 5. Figure 5A shows that there are no significant changes on the surface of the modified electrode after 12 measurements. Still, slight changes on the Nafion/MWCNT film are observed after 20 measurements on the cross section image of Figure 5B, being possible to observe the presence of CNTs under the Nafion layer, and after 50 determinations only some very tiny deformations are observed. It is interesting to note that despite their disposability, the developed Nafion/MWCNT-SPCEs can be used at least in 50 consecutive determinations without loss of the major physical properties of its surface, which is in agreement with the voltammetric results.

**Figure 6:**
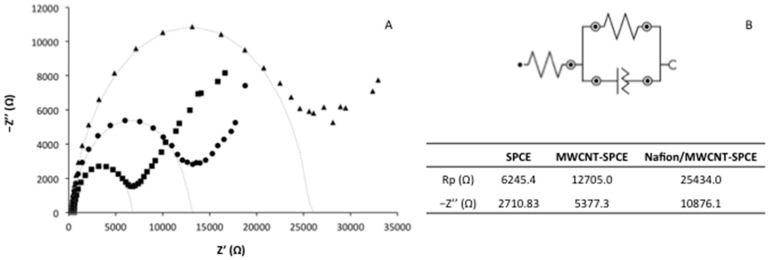
(**A**) EIS measurements of a 2 mM solution [Fe(CN)_6_]^3−/4−^ at unmodified-SPCE (squares), MWCNT-SPCE (circles) and 0.0250% Nafion/MWCNT-SPCE (triangles); Dashed lines correspond the electrochemical circle fitting data; (**B**) Equivalent circuit model and EIS parameters found from the experimental data fitting and simulation of the equivalent circuit model for SPCE, MWCNT-SPCE and Nafion/MWCNT-SPCE.

Attempting to understand the physical implications of the different modifiers applied, the impedance changes on the electrodes surfaces was evaluated through EIS measurements in the presence of equimolar [Fe(CN)_6_]^3−/4−^ ions. Impedance spectroscopy under open circuit potential (OCP) was carried out by applying a sine wave of 0.01 V amplitude over a frequency range of 100 kHz to 0.1 Hz. Nyquist plots of unmodified-, MWCNT- and Nafion/MWCNT-SPCEs are shown in Figure 6A. In all instances, EIS graphs of the imaginary impedance (−*Z*’’) versus real impedance (*Z*’) show two well-defined regions: a semicircle in the high frequency range, that corresponds to the [Fe(CN)_6_]^3−/4−^ redox couple charge transfer process through the electrode surface; and a linear region in the low frequency range, characteristic of the Warburg impedance, that corresponds to the diffusion controlled process within the electrode [43].

It can be seen that the electrical impedance of the system strongly depends on the modifier layers employed, being found that the addition of each coating material increases both imaginary and real impedance. Figure 6B depicts the equivalent circuit applied and the results from the simulation experiments for SPCE, MWCNT-SPCE and Nafion/MWCNT-SPCE. Indeed, the charge transfer resistance is higher when the carbon nanostructures are added to the carbon electrode surface. Similarly, the Nafion polymer film addition increased the impedance of the electrochemical system. EIS results show that the diameter of the semicircle increases with the addition of both coating layers, allowing the confirmation of their presence on the carbon electrodes surface, which is in agreement with our expectations [44] and demonstrates the utility of this method on controlling the coating procedure, complementing the results reported above.

### 3.3. Electrochemical Behaviour of ETB

#### 3.3.1. Influence of pH

**Figure 7:**
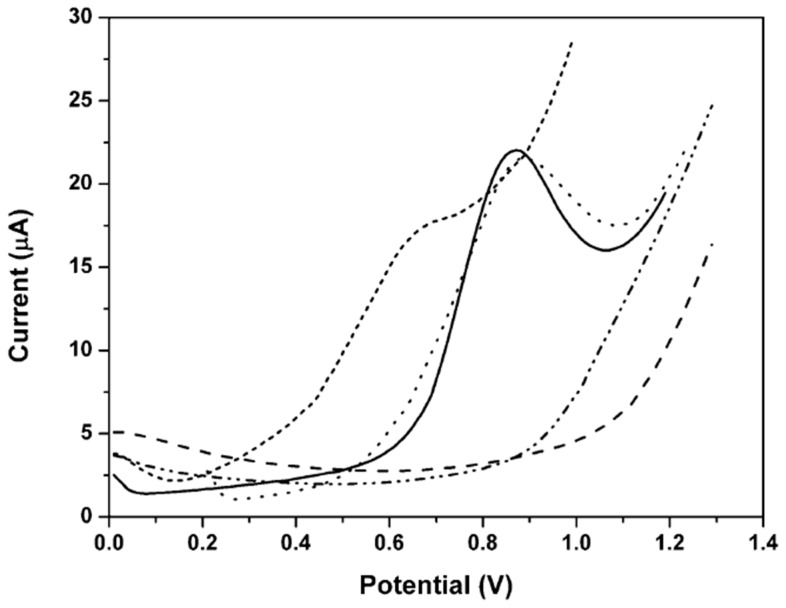
Square wave voltammograms of a 0.14 mg/μL ETB solution at pH 2.0 (dash line), 4.0 (dash dot dot line), 7.4 (solid line), 9.0 (dot line) and 11.0 (short dash line) at the surface of 0.0250% Nafion/MWCNT-SPCE. Frequency: 10.0 Hz; Potential Step: 0.01 V; Potential Amplitude: 0.01 V.

The influence of pH on the electroxidation behaviour of ETB at the surface of the Nafion/MWCNT-SPCEs was evaluated by SWV at different pH values, ranging from 2 to 11. Figure 7 depicts the square wave voltammograms obtained for a 0.28 mg/μL ETB solution at pH 2.0, 4.0, 7.4, 9.0 and 11.0, being observed that the analytical response, by means of *I_pa_* and *E_pa_* parameters, is highly dependent on the pH value of the drug solution. Actually, at pH 2 no anodic peak is observed and at pH 4 a very poor signal is found. By contrast, well-defined oxidation waves are obtained at pH values greater than 7, being observed a displacement into more anodic potentials with the increasing of pH. As it can be confirmed by the square wave voltammograms between 0 and 1300 mV, the best oxidation signal was obtained at CPBS pH 7.4, which was selected for the following studies. This comportment is in agreement with the initially predicted Nafion selectivity for the drug at physiological pH.

#### 3.3.2. Oxidation Behaviour Analysis 

**Figure 8:**
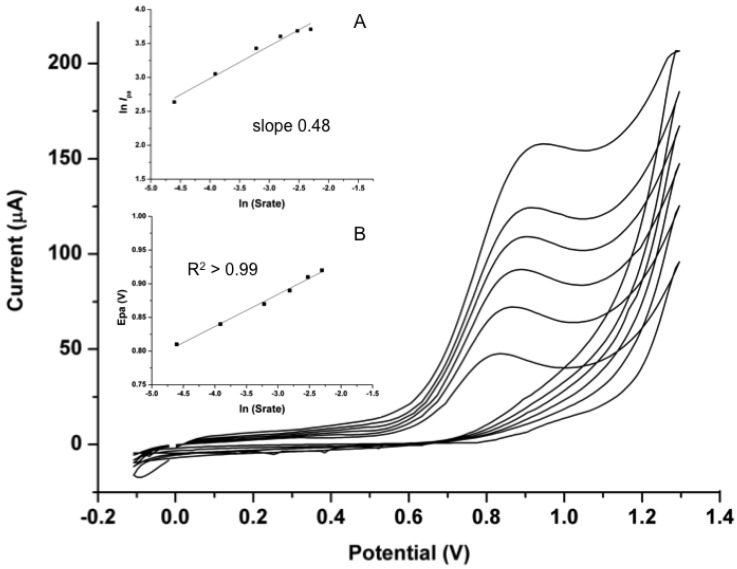
Cyclic voltammograms of a 0.14 mg/μL ETB solution in CPBS pH 7.4 on a 0.0250% Nafion/MWCNT-SPCE at different scan rates, from 0.01 to 0.2 V·s^−1^. Inset A: napierian logarithm of oxidation peak current versus napierian logarithm of scan rate; Inset B: oxidation peak potential versus napierian logarithm of scan rate.

The electrochemical behaviour of ETB was studied through CV at pH 7.4 at the optimized electrodes, i.e., 0.025% Nafion/MWCNT-SPCEs, between −100 and 1300 mV. ETB *I*_pa_ was measured as a function of the potential scan rate. Figure 8 shows a serie of cyclic voltammograms of a 0.14 mg/μL ETB solution at scan rates from 0.01 to 0.2 V·s^−1^ in CPBS pH 7.4. Cyclic voltammograms of the drug gave one well-defined anodic peak that from comparison with the literature can be ascribed to the oxidation of the alcohol moieties of the ETB molecule [45]. There is no peak on the reverse scan, indicating the irreversibility of the process. This study revealed that the *I*_pa_ increases linearly with the square root of the scan rates (*v*^1/2^), suggesting that ETB follows a diffusion controlled oxidation process. Moreover, the correlation between the Napierian logarithm of the scan rate and the napierian logarithm of the *I*_pa_ was found to yield a straight line of slope 0.48, which is close to the expected value of 0.5 for diffusional species (inset A of Figure 8). Also, it was observed that the *E*_pa_ shifts into more positive values with increasing of the scan rate, with a good linear dependence of the *E*_pa_ upon the napierian logarithm of the scan rate (ln*v*) (inset B of Figure 8), which confirms the irreversibility of the process. The outcomes of this experiment suggest that the oxidation of this antimycobacterium agent at the surface of the obtained Nafion/MWCNTs modified electrodes undergoes a typical diffusion controlled electrochemical process. 

#### 3.3.3. Square Wave Voltammetry

**Figure 9:**
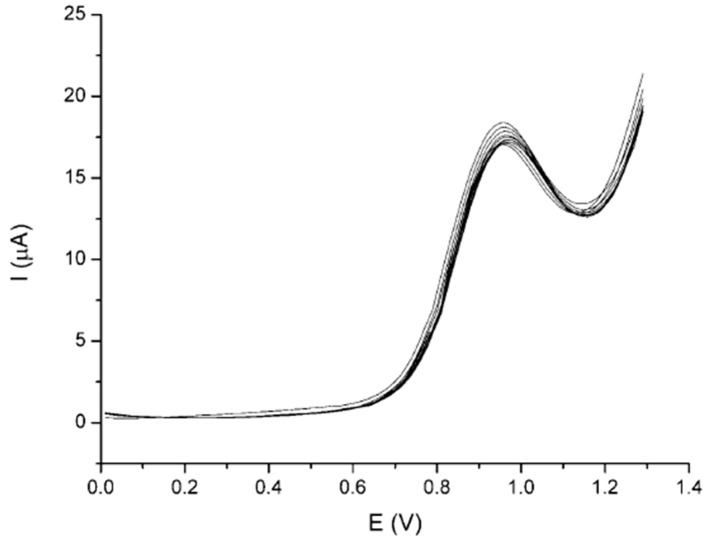
Series of ten square wave voltammograms of a 0.14 mg/μL ETB solution in CPBS pH 7.4. Frequency: 10.0 Hz; Potential Step: 0.01 V; Potential Amplitude: 0.01 V.

To apply the developed Nafion/MWCNT-SPCEs to the electrochemical sensing of the first line anti-TB compound ETB, SWV technique was employed. As such, SWV parameters were optimized, namely the frequency (1.0, 2.0, 4.0, 6.0, 8.0, 10.0, 12.0 and 15.0 Hz), the potential step (*E*_step_) (0.005, 0.010 and 0.020 V) and the potential amplitude (*E*_amp_) (0.0025, 0.0050, 0.0075, 0.0100, 0.0125, 0.0150, 0.0300, 0.0400, 0.0600, 0.0800 and 0.1000 V) in order to obtain the greatest oxidation peak definition associated with the highest *I*_pa_. The experimental parameters were established as 10.0 Hz frequency, 0.01 V *E*_step_ and 0.01 V *E*_amp_. Using these conditions, several concentrations of ETB were analyzed at the surface of Nafion/MWCNT-SPCEs and a calibration curve over a range of 0.028 to 0.28 mg/μL was obtained, which fitted the equation *I*_pa_ (μA) = 1.782 + 25.37 × C_ETB_ (mg/μL), *r*^2^ > 0.99. Limits of detection (three times the standard deviation of the intercept/slope) and quantification (ten times the standard deviation of the intercept/slope) of 8.4 × 10^−4^ mg/μL and 2.8 × 10^−2^ mg/μL, respectively, were achieved. Repeatability and reproducibility studies of the 0.025% Nafion/MWCNT-SPCEs were performed using a 0.14 mg/μL ETB solution. The repeatability was evaluated by measuring ten consecutive square wave voltammograms (Figure 9) under the optimized conditions and the reproducibility was estimated using three 0.025% Nafion/MWCNT-SPCEs prepared under the same conditions. Standard deviations of 4.3% (*n* = 10) and 1.9% (*n* = 3), respectively, were registered, which is in agreement with the observations from SEM images and confirms the appropriateness and reliability of the proposed approach. 

### 3.4. Analytical Application to Biological Analysis 

Very few works reporting the electrochemical determination of ETB have been published. Among them, Lima et al. proposed a gold-microelectrode array with a LOD of 1.55 × 10^−4^ mM, which was only applied to aqueous solutions [45]. Shao et al. applied a MWCNT modified glassy carbon electrode to the detection of this drug in pharmaceutical formulations with a LOD of 7.6 × 10^−4^ mM [46]. Perantoni et al. reported two graphite-based electrodes for the amperometric detection of ETB in pharmaceutical formulations [41] and in synthetic urine [47] with detection limits of 0.1 mM and 0.0634 mM, respectively. A biosensor based on the use of silver nanoparticles was developed by Ngece et al., exhibiting a LOD of 7.0 × 10^−4^ mM ETB in serum samples [40]. 

In order to evaluate the applicability of the proposed Nafion based-sensor, the determination of ETB in biological matrixes (i.e., human urine and serum samples) was attempted. To this end, SWV was performed under the optimal conditions. Human urine and blood serum samples were firstly spiked with final ETB concentrations of 1.4 mg/μL and 0.84 mg/μL, respectively. Then, the samples were diluted ten and two times, respectively, with CPBS pH 7.4, in order to achieve the concentrations of 0.14 and 0.42 mg/μL. Biological specimens weren’t subjected to additional pre-treatment steps and SWV measurements were performed in triplicate with a 100 μL drop of each sample. The proposed analytical method allowed the detection of ETB in both samples with great precision and accuracy and relative errors of 1.9% and 2.9% (*n* = 3), respectively for human urine and blood serum samples.

Giving the complexity of both human urine and blood matrixes and the no need of pre-treatment steps reported herein, such results indicate the suitable performance of the proposed modification procedure on the development of this Nafion-MWCNT-SPCE-based electrochemical sensing interface for the ETB sensing. The outcomes of this paper are strongly encouraging, providing the basis for the design of a portable and reliable electrochemical strip test for this first line antimycobacterial drug.

## 4. Conclusions

In this work, we have successfully developed a rapid, sensitive and cost effective portable sensor for the biological sensing of ETB in small volumes of samples based on the use of modified and disposable SPEs. The developed Nafion/MWCNT-SPCEs showed high sensitivity and selectivity for the analysed drug; indeed, negatively charged character of Nafion strongly attracts the anti-TB drug ETB, which is a cationic antimycobacterial agent at physiological pH, and considerably minimized the influence of matrixes interferences. The proposed electrodes greatly improved the oxidation analytical signal of ETB compared with both MWCNT- and unmodified-SPCEs, showing a LOD of 8.4 × 10^−4^ mg/μL and satisfactory repeatability and reproducibility. Results demonstrate that the use of Nafion/MWCNT-SPCEs can be exploited as electrochemical devices for ETB sensing, being a promising approach that provides a low-cost, portable yet highly reproducible and reliable alternative platform for the ‘at-point-of use’ detection of this anti-TB agent.
